# Amino Acids Influencing Intestinal Development and Health of the Piglets

**DOI:** 10.3390/ani9060302

**Published:** 2019-05-31

**Authors:** Qi Mou, Huan-Sheng Yang, Yu-Long Yin, Peng-Fei Huang

**Affiliations:** School of Life Sciences, Hunan Normal University, Changsha 410006, China; mouqi@foxmail.com (Q.M.); yhs@hunnu.edu.cn (H.-S.Y.); yinyulong@isa.ac.cn (Y.-L.Y.)

**Keywords:** amino acid, intestinal development, piglet health

## Abstract

**Simple Summary:**

The health of piglets is an important issue in pig production. Nutritional support for intestinal development is a significant component of piglet care, and amino acids are essential for intestinal growth and development. For suckling piglets, the sows’ milk and the maternal environment shape the structure and support the function of the intestinal tract. The composition of milk affects intestinal morphology and the digestive, absorption and barrier function. After weaning, the optimal nutritional strategies of their diet are necessary to guarantee the piglets’ intestinal development and growth performance. Amino acids are the most important ingredient in piglet diets. The aim of this review is to collect and analyze the relationship between amino acid nutrition and intestinal development of piglets, and elucidate the impacts on piglet health.

**Abstract:**

The amino acids and other components of diet provide nourishment for piglet intestinal development and maturation. However, early-weaned piglets struggle with tremendous stress, impairing normal intestinal health and leading to intestinal dysfunction and even death. The high prevalence worldwide of post-weaning diarrhoea syndrome (PWDS) in piglets has led to much interest in understanding the important role of nutrients in the establishment and maintenance of a functional intestinal tract. In particular, the impacts of amino acids on these functions must be considered. Amino acid levels greatly influence intestinal development in weaning piglets. The lack of amino acids can cause marked structural and functional changes in the intestine. Therefore, a comprehensive understanding of the functions of amino acids is necessary to optimize amino acid requirements of the developing intestinal tract to maximize piglet health and growth performance. This review summarizes the role of specific amino acids (arginine, glutamate, threonine, sulphur-containing amino acids (SCAAs), and branched-chain amino acids (BCAAs)) that have been proven to be beneficial for the intestinal health of weaned piglets.

## 1. Introduction

Intestinal development disorder is one of the most important causes of piglet morbidity and mortality during the immediate post-weaning period [1]. At weaning, there are significant changes of nutritional supplies in piglets. That is to say, the piglet has to deal with the indigestible diets to maintain the high rate of intestinal structure formation and functional maturation [2]. Intestinal epithelial cells and the immune system are both of vital importance for digestion, absorption, barrier function and homeostasis in the body. The complex interactions occurring in the intestine between nutrients and the mucosal epithelium play very important roles in piglet health maintenance and/or regulation [3,4,5]. Malnutrition could lead to morphological and functional changes in the intestine. Due to the adverse influences on intestinal morphology after weaning, intestinal functions can be incomplete, resulting in decreased digestive, absorptive, barrier and immune capacities [6]. These functions have a direct effect on intestinal health in piglet husbandry practices. To minimize the adverse impacts of weaning and their subsequent consequences, appropriate nutritional strategies must be considered to maximize the growth performance of weaned piglets [7,8,9]. Therefore, the development of normal intestinal structure and function is a vitally important determinant of piglet health, and subsequently in reducing incidences of diarrhoea and increasing growth performance. Luminal nutrient supply is a critical component in the establishment of integral structure and homeostatic function of intestine.

Adaptation to the dry diets containing complex components is the first challenging factor faced by the piglets after weaning, because most nutrients are supplied via the sows’ milk before weaning [10,11]. Nutrients in the solid diets stimulate the further development of the intestines [12]. The deficiency of essential nutrients could result in intestinal dysfunction, diarrhoea, growth performance disease and even death [13,14]. Although piglet intestinal diseases are understood throughout the world, the pathomechanisms associated with the impacts of nutrition are not well recognized. Furthermore, the influence of dietary components on the developing intestinal tract is an important area of much research emphasis to protect piglet health and improve growth performance [15,16,17]. Animal nutritionists are committed to optimize nutrient requirements, and amino acids are the most commonly affected nutrients of weaned piglets [18,19,20,21]. However, a better comprehension of the individual amino acids is needed for piglet nutritional requirements in husbandry practices. The objective of this paper is to review the contributions of various amino acids in support of optimal intestinal development for piglet health, especially for weaned piglets.

## 2. Impacts of Amino Acids on Piglet Intestinal Health

The piglet intestinal tract requires nutritional support for its growth and development as well as the function of digestion, absorption and the mucosal barrier [22,23]. In recent years, amino acids have been recognized to be important nutrients that promote intestinal development and health of piglets. In order to obtain optimal function of the intestine, long villi are needed [24]. However, diets with high levels of soybean with strong anti-nutritional factors may cause villi to atrophy. Lower dietary protein levels have been shown to be a nutritional strategy to improve the intestinal structure and function of weaned piglets, but may result in insufficient amino acid supply and impaired piglet growth performance [25]. Therefore, it is necessary to balance the crystalline amino acids in a low protein diet to avoid amino acid deficiency.

Dietary amino acids elicit a variety of nutritional and metabolic functions. Nutritionally, amino acids are classified as essential or nonessential for animals based on their traditional role in protein synthesis. However, the critical regulatory roles for amino acids in metabolism have long been ignored. In fact, amino acids and their metabolites are regulators of cellular signal transduction, gene expression and the protein post-translational modification, especially in the intestine [26,27]. The optimal balance between amino acids in diet and circulation is essential for systemic homeostasis and adequate amino acid intake is especially important for the intestinal physiology of weaned piglets [20,28]. Thus, we are concerned about the latest advances regarding the influences of individual amino acids on intestinal development and weaned piglet health.

### 2.1. Arginine

The abundance of arginine in pig tissues is very high, which plays an important role in regulating gene expression and cell signaling [29]. Adding adequate arginine to the diet can significantly improve the growth, lactation and reproductive performance of swine [30]. According to the traditional nitrogen balance experiment results, it is believed that piglets can synthesize a sufficient amount of arginine to meet their physiological needs [31]. With the advancement of breeding technology, new breeds of pigs have stronger growth potential, more lean tissue, and can breed more fetuses. Therefore, modern breeds require more arginine than in the past. However, current low protein diets may not provide sufficient arginine for pig growth, especially affecting intestinal development, survival and growth performance in weaned piglets.

Current researches have recognized that arginine is a conditionally essential amino acid for pigs at all stages of pig production [32,33]. Adding arginine to the basal diet can improve the growth performance of modern breeds of pigs [34]. Animal nutritionists have proposed higher dietary arginine requirements to maximize pig growth, sow milk production and fetal survival [35]. The piglets are very sensitive to the supply of dietary arginine, and the severe lack of arginine will quickly lead to hyperammonemia and even death [36]. Because sow milk is relatively deficient in arginine, supplementation of arginine to piglets can significantly increase serum arginine concentration, reduce ammonia levels and increase piglet weight [37]. Interestingly, supplementation with arginine before weaning can enhance intestinal growth and development after weaning [38]. These data further demonstrate that sufficient arginine in the diet is necessary to ensure maximum growth performance of the piglets. Piglet feed intake was significantly reduced after weaning, and the typical diet had lower arginine content. Piglets can effectively use citrulline in the diet to synthesize arginine. Therefore, adding arginine or equivalent citrulline to the weaning diet can increase the concentration of arginine in the serum and the growth performance of the pig. The addition of arginine to the basal diet after weaning can increase the weight and development of the intestine, which is important for improving the health of piglets.

### 2.2. Glutamate

Glutamate is considered to be a non-essential amino acid, but it plays an important role in maintaining the growth and health of piglets [39,40]. Glutamate is a highly abundant free amino acid in milk and cells and is a key regulator of gene expression and cell signaling [41,42]. However glutamate may be limited when feeding low protein diets during pregnancy, lactation and the weaning period [43]. For instance, dietary supplementation with glutamate prevents the loss of sow weight during lactation and increases glutamine content in milk [44]. The addition of glutamate to the diets of suckling and weaning pigs significantly improves intestinal morphology and immune function, thereby promoting growth performance [45]. In addition, dietary supplementation with glutamate can reduce skeletal muscle loss in pigs caused by endotoxin challenge [46]. Therefore, the addition of sufficient glutamate to the diet is important for improving the nutrition and health of piglets.

Glutamate is one of the most abundant amino acids in food and animal tissues, accounting for 5% to 15% of dietary protein [26]. Due to the enormous role of glutamate in metabolism and physiology, it has received increasing attention from nutritionists. In particular, glutamate has recently been identified as a nutritionally essential amino acid in the intestinal and systemic homeostasis of piglets, and studies on the nutritional requirements for glutamate in weaned piglets are increasing [45]. Supplementation of glutamate in low protein diets can increase the daily gain of weaned piglets without affecting feed conversion ratio [47]. This suggests that low protein diets do not provide enough glutamate to weaned piglets. Similarly, the addition of arginine to weaned diets significantly increased feed intake and daily gain in piglets [48]. Intestinal dysfunction in weaned piglets is a major concern for pig producers worldwide. In order to achieve optimal intestinal health and growth, weaned piglets should receive at least the amount of glutamate corresponding to lactation. However, compared to suckling piglets, the feed intake in weaned piglets during the first week after weaning usually decreases rapidly. Therefore, the weaning piglet diet can only provide half of the amount of amino acids required for piglet intestinal development. Obviously, dietary supplementation with glutamate is essential for the health of weaned piglets, which is especially important for piglets fed a diet contaminated with mycotoxins [49,50]. According to the results of the study, supplementation with glutamate after weaning increased the height of the intestine villi, and increased the ability of epithelial cells to proliferate and differentiate and resist oxidation. Moreover, dietary glutamate dose-dependently reduced the incidence of post-weaning diarrhea [49,50]. These data suggest that glutamate can improve the intestinal health and the growth performance of weaned piglets.

### 2.3. Threonine

In pig production, low protein diets not only reduce farming costs, but also reduce environmental pollution. Replacing crude protein with crystalline amino acids can reduce dietary protein levels without compromising the growth performance of pigs [51,52]. Threonine is the second limiting amino acid in pig diets, which plays a key role in maintaining intestinal mucosal integrity and barrier function in piglets [53]. The addition of threonine to the diet reduces digestive enzyme production and increases intestinal pericellular cell permeability [54]. Most dietary threonine is used for intestinal mucosal protein synthesis, especially for mucin synthesis [55]. Since mucin is not digested and re-used by the intestine, intestinal mucin secretion is a net loss of threonine. The content of threonine in the intestinal lumen can affect the synthesis of intestinal mucin and other proteins. Therefore, the demand for threonine in weaned piglets will increase to maintain the functions of the intestine. Threonine is essential for improving intestinal structure and morphology in piglets under physiological and pathological conditions.

Many previous studies have focused on the requirement, efficacy and metabolism of threonine [56]. Recently, many researchers have reported the relationship between the threonine metabolism and intestinal health in piglets [57]. Weaned piglets need enough threonine to support their optimal growth performance. However, excessive or lack of threonine can reduce feed intake and growth rate, and impair immune function [58]. The intestine has multiple functions, such as digestion and absorption of nutrients, as well as immune defenses against pathogens and toxins [59]. Intestinal functions are largely dependent on the integrity of the morphological structure, which protects the organism from harmful substances [60]. Many factors can affect intestinal mucosal integrity. Among these factors, threonine has the most profound effects on intestinal mucosal barrier function and is essential for animal and human health. Intestinal mucus acts as a physical barrier against pathogen invasion. The amount of mucus produced by goblet cells is an important indicator for assessing intestinal integrity. The actual mucus production is difficult to measure directly and can be estimated indirectly by the number of goblet cells.

### 2.4. Tryptophan

Tryptophan is one of the essential amino acids in pigs, and is also the main protein component of animals. Tryptophan has many physiological and biochemical functions during the growth and development of pigs. Although researches on the functions and applications of tryptophan have been ongoing, it is currently not widely used in livestock production due to the high cost of synthesis. Moreover, there are many studies on the applications of tryptophan to livestock and poultry farming, but it is difficult to exert the potential application value of tryptophan because of the lack of systemicity.

As an essential amino acid in pigs, tryptophan plays an important role in immune regulation, digestion regulation, anti-stress and protein synthesis regulation. In the production of pigs, the addition of tryptophan can improve the growth performance of growing-finishing pigs and ameliorate the health of weaning piglets. Weaning of piglets may result in decreased daily feed intake, growth performance, gastrointestinal health and immune function, which may lead to susceptible diseases. Studies have shown that feeding the weaned piglets with tryptophan diets significantly reduced the frequency and duration of struggles between the piglets, and improved the integrity of the intestinal morphology [61]. Moreover, the concentration of cortisol in the saliva of piglets was increased, while the concentrations of norepinephrine and adrenal gland were decreased by the addition of tryptophan [62]. In the future, in addition to continuing research on the physiological and biochemical functions of tryptophan, the synthesis process of tryptophan should be further optimized to reduce production costs. In addition, systematic studies should be carried out on the appropriate amount of tryptophan in pigs at different growth stages to accumulate rich data and provide technical support for its wide applications.

### 2.5. Sulphur-Containing Amino Acids

The metabolism of sulphur-containing amino acids (SCAAs) plays a key role in ensuring cell functions. In addition to their role in protein biosynthesis, SCAAs are important biologically active molecules. SCAAs can affect cellular metabolism and function by participating in the regulation of methylation processes and redox states [63]. The important functions of SCAAs in the diet have been extensively studied, and the lack of sulphur amino acids can lead to a variety of systemic dysfunctions [64]. After weaning, there are three main reasons for the lack of SCAAs in piglets: (1) The content of SCAAs in the diet cannot meet the growth and development requirements of piglets; (2) feed intake of piglets is drastically reduced, resulting in insufficient amino acid intake; (3) increased SCAAs catabolism caused by resistance to weaning stress, resulting in insufficient SCAA availability. Therefore, it is important to determine the appropriate intake of SCAAs and provide a justification for the SCAA requirements of weaned piglets [64,65,66,67].

More importantly, the SCAAs in the diet are critical to the normal functions of the intestinal tract [68]. The intestine is an important organ for the metabolism of SCAAs in the body. About 20% of the SCAAs in the diet are transmethylated into cysteine and homocysteine in the intestine [69]. Cysteine is the rate-limiting amino acid of glutathione synthesis and is the main cellular antioxidant in mammals [70]. As a precursor of glutathione, cysteine in the diet plays a key role in the antioxidant function of the intestinal epithelium [71]. Moreover, cysteine and glutathione can also regulate epithelial cell proliferation and differentiation by regulating the redox state [68]. Further research is needed to determine how SCAAs regulate intestinal growth and intestinal function in piglets and contribute to the development of the gastrointestinal tract.

### 2.6. Branched-Chain Amino Cids

The branched-chain amino acids (BCAAs) are essential substrates for protein biosynthesis. In addition, recent studies have shown that BCAAs are involved in intestinal growth and development [72]. Dietary supplementation of BCAAs promotes intestinal digestive enzyme activity, epithelial nutrient transporter expression, barrier function and the production of beneficial intestinal bacteria of piglets [73,74]. The regulation of the expression of genes and proteins involved in signaling pathways is a potential mechanism for the action of BCAAs [75]. Convincing evidences support this view: BCAAS, as functional amino acids with various physiological roles, play a key role in promoting intestinal development and piglet health [72,76,77]. 

As functional amino acids, BCAAs participate in and regulate key metabolic pathways to improve intestinal development and systemic growth in weaned piglets. BCAAs have a protective effect on intestinal redox status and related gene abundance in weaned piglets with growth retardation [78]. Dietary BCAA supplementation could regulate energy metabolism in pig intestinal epithelial cells by activating the different pathways, thereby reducing harmful species levels [79]. Dietary supplementation with BCAAs reduces intestinal disease in weaned piglets caused by pathogens [80]. Additionally, the BCAA supplementation in the diet reduces immune system stimulation during weaning, but does not affect the systemic protein nutrition of the piglets [81]. Dietary supplementation with BCAAs can improve the survival rate of premature piglets and improve the growth performance during weaning [82]. The integrity of intestinal morphology is critical for intestinal functions, and BCAAs can play an important role in the maintenance of intestinal structure. It has been reported that dietary BCAAs have a positive effect on the proliferation and differentiation of piglet intestinal epithelial cells [79]. Energy supply is important during piglet intestinal development. Glutamate and aspartate are the main energy sources for intestinal digestive function and intestinal mucosal integrity of piglets. BCAAs provide amino groups for glutamate and aspartate synthesis in the intestine, and also directly provide energy for nutrient transport and intracellular protein turnover [83,84]. In conclusion, the addition of appropriate BCAAs to the diet is beneficial to the intestinal development and health of the piglets.

## 3. Conclusions

Understanding the dependence of intestinal development on nutrients can help nutritionists to provide optimal nutritional support for weaned piglet health. Amino acids are important functional nutrients involved in the maintenance of the morphology and functions of intestine. Researches on the nutritional and physiological functions of essential amino acids have been very in-depth, and there are suitable recommended additions for piglets. Among them, the recommended level of lysine is 1.45%, arginine is 0.68%, threonine is 0.88%, tryptophan is 0.25%, SCAAs are 0.79% and BCAAs are 1.32% [85]. Intestinal diseases caused by weaning of piglets are the main cause for the loss of pig production. At present, antibiotics are still the main means of preventing and treating weaning stress syndrome. In order to minimize the impact of antibiotic removal on weaned piglets, it is necessary to develop effective antibiotic alternatives. Feeding a low protein diet with crystalline amino acids after weaning is one strategy which can improve the intestinal development and health of piglets.
